# Quantitative Linking Hypotheses for Infant Eye Movements

**DOI:** 10.1371/journal.pone.0047419

**Published:** 2012-10-26

**Authors:** Daniel Yurovsky, Shohei Hidaka, Rachel Wu

**Affiliations:** 1 Department of Psychology, Stanford University, Stanford, California, United States of America; 2 School of Knowledge Science, Japan Advanced Institute of Science and Technology, Nomi, Ishikawa, Japan; 3 Department of Brain and Cognitive Sciences, University of Rochester, Rochester, New York, United States of America; Kyushu University, Japan

## Abstract

The study of cognitive development hinges, largely, on the analysis of infant looking. But analyses of eye gaze data require the adoption of linking hypotheses: assumptions about the relationship between observed eye movements and underlying cognitive processes. We develop a general framework for constructing, testing, and comparing these hypotheses, and thus for producing new insights into early cognitive development. We first introduce the general framework – applicable to any infant gaze experiment – and then demonstrate its utility by analyzing data from a set of experiments investigating the role of attentional cues in infant learning. The new analysis uncovers significantly more structure in these data, finding evidence of learning that was not found in standard analyses and showing an unexpected relationship between cue use and learning rate. Finally, we discuss general implications for the construction and testing of quantitative linking hypotheses. MATLAB code for sample linking hypotheses can be found on the first author's website.

## Introduction

The study of infant cognitive development hinges largely on the analysis of infant looking behavior [1]. Since Fantz's [2] landmark demonstration of visual memory in 2-month-old infants, researchers have used his habituation technique, and other eye-movement methods, to ask deep theoretical questions about the ontogeny and development of human cognition. But analysis of eye-movements, like analysis of other high-dimensional cognitive measures (e.g. fMRI, EEG) carries particular challenges [3]. In order to connect observed eye-movements to underlying cognitive processes, one must define a *linking hypothesis* that relates them [1], [4].

Every experimental paradigm used in the study of infant cognition commits – even if only implicitly – to a particular *linking hypothesis*. For instance, in habituation studies [2], [5], decreased looking is hypothesized to indicate encoding, and recovery from decreased looking is hypothesized to indicate discrimination of a novel stimulus from a previously encoded stimulus. In violation of expectation studies [6], [7], increased looking is hypothesized to indicate noticing a surprising event. In intermodal preferential looking studies [8], [9] a difference in looking time to one sound-object mapping over another is hypothesized to indicate a difference in their associations. But, critically, all of these linking hypotheses are qualitative; they assert that a relationship exists but do not specify its *quantitative*, metric properties.

Why should we prefer quantitative linking hypotheses? Quantitative linking hypotheses are important for moving from asking *if* a phenomenon occurs to asking *how* and *why*. First, quantitative linking hypotheses allow researchers to clearly and unambiguously specify the assumptions and mechanisms in their theories. As theories grow in complexity, correctly deriving their (sometimes counterintuitive) predictions can become quite difficult. Formalizing these theories makes such predictions tractable [10], [11]. Second, without quantitative linking hypotheses, it can often be impossible to distinguish competing theoretical accounts of the data in a given experiment. This problem has fueled many debates among developmentalists about whether eye-movement patterns observed in a given experiment are best given “rich” (conceptual) or “lean” (perceptual) theoretical explanations [12]–[14]. Third, quantitative linking hypotheses allow researchers to test the same theoretical model *across* experiments, integrating multiple datasets within one self-consistent framework [1], [14]–[16]. The memory [17], vision [18], and cognitive architecture [19] literatures provide excellent examples of the importance of this kind of theory building, which has remained elusive in the developmental literature (although, see [20]).

Developmentalists who measure eye-movements face several challenges in the construction of quantitative linking hypotheses. First, control of eye-movements is complex, and saccades are likely to be moderated by multiple systems [1], [21], [22]. Even in viewing natural scenes, for instance, fixation patterns are moderated not only by multiple components of visual salience [18], but also by higher-order scene statistics [23] and task goals [22], [24]. Quantitative linking hypotheses, then, must be capable of dealing with multiple interacting components.

Second, when fixation duration is used as an indication of learning, as in many preferential looking paradigms, it is unclear whether their relationship is a simple linear one. For instance, some experiments may find a robust novelty preference, while others find a robust familiarity preference in a similar paradigm [25], [26]. A number of authors have consequently proposed that learning and looking may be linked non-monotonically, with a preference for familiarity appearing first, and a preference for novelty developing with further experience [27]–[30]. Any linking hypothesis used in such paradigms must be flexible enough to accommodate this kind of complexity.

Third, while all experimental psychologists must contend with variability among participants, for developmentalists this problem is particularly pronounced. Development, especially during infancy, is a time of rapid change, and two participants at the same age may be at markedly different points in their developmental trajectories. Thus, the same linking hypothesis may not be appropriate for all infants. While the issue of averaging over qualitatively different types of participants is well-known in both the adult [31], [32] and developmental [33] literatures, it is rarely tackled directly. When it is, researchers typically perform a median split on the measure of interest to accommodate individual differences [34], [35]. But one cannot know apriori whether the data is best analyzed as one group, or two, or three or more. A system for generating and testing quantitative linking hypotheses must be able to deal gracefully with this kind of complexity.

This paper proposes a framework for the construction and analysis of quantitative linking hypotheses for data from eye gaze experiments. We build on a growing body of statistical tools – non-parametric Bayesian models – to produce a principled, rigorous, empirically successful analysis that meets the challenges reviewed above. This framework allows linking hypotheses to be composed of multiple interacting components, for each of these components to have any functional form, and for qualitatively different groups of infants to be automatically and adaptively identified. To demonstrate the utility of this framework, we analyze data from a set of experiments investigating the role of social and non-social cues in infant multi-modal learning [36]. This analysis shows how quantitative linking hypotheses provide leverage in understanding the development and operation of infant learning mechanisms. We show that, across conditions, infants cluster coherently into several different types of learners, that these different types of learners are affected differently by the presence of a social cue, and that the non-social cue impairs learning by competing for attention. We conclude with a discussion of how this framework could be extended to deal with other kinds of data, to compare competing theories within an experiment, and to aggregate data across experiments.

The rest of the paper is organized as follows. First, we describe the general framework for the construction and analysis of quantitative linking hypotheses. Next, we present a specific instantiation of this framework constructed to analyze a set of studies investigating the relationship between attentional cues and learning in 8-month-old infants [36]. Third, in order to empirically validate the framework, we show that this analysis performs as expected in a set of simulation studies comparable to those in which the infants participated. Fourth, we apply the analysis to empirical data and show how this novel framework provides insight into cognitive processes that was unavailable in the standard analyses. Finally, we conclude with a discussion of how this analysis can be applied and extended for use in other infant experiments, and how it can be used to discriminate among competing theories.

## Analysis

### General Framework

We begin by describing the framework in which we propose to use quantitative linking hypotheses to analyze infant eye movement experiments. Here we describe, at a conceptual level, how these tools meet the challenges reviewed above, and how their output can be interpreted. Full technical details can be found in Graphical Model Details S1.

Consider a typical infant eye-tracking experiment. In such an experiment, each infant is exposed to stimuli that encode some structure of theoretical interest. The researcher measures this structure's influence on infants' looking behavior. For instance, in studies of early numerical cognition, researchers expose infants to displays of dots varying along a number of dimensions (e.g. cumulative contour, area, etc.), but consistent in one: number of dots [37], [38]. Consistent structure along this one dimension subsequently leads infants to prefer displays of a different number. In studies of categorization, infants are exposed to visual objects that vary along many dimensions, but are consistent in dimensions that define a particular category [39], [40]. This consistency in structure leads infants to look longer at objects from a different category. In infant word-learning experiments, infants are exposed to consistent pairings between words and objects [9], [41]. Infants subsequently discriminate between word-object mappings consistent with training stimuli, and those that are inconsistent. In all of these cases, however, the researcher is not interested directly in the change in the observed looking behavior, but rather in the cognitive processes it implicates [42], [43]. Quantitative linking hypotheses let us describe these processes directly.

For each infant on each trial, the researcher observes some eye-gaze data (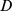). The researchers goal is to determine the model (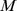) that is most likely given the observed eye movements (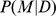). This problem can be formalized as a problem of Bayesian inference. The researcher can specify several possible models, each of which makes different predictions about the gaze data likely to be observed (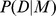). The researcher may also prefer simpler models apriori in accord with Ockhams razor (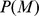). These properties can then be combined via Bayes' rule to infer the model that best describes the infants cognitive processes (Equation 1).
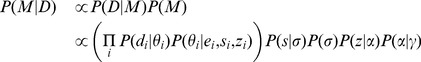
(1)


**Figure 1:**
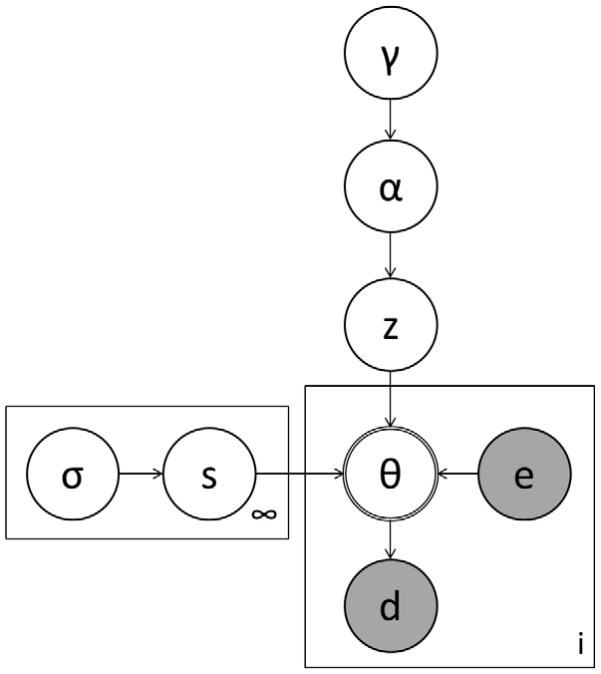
Graphical Model for Infant Eye Movements. A graphical model for inferring the cognitive processes (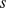) responsible for generated eye movements (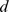) under particular experimental conditions (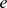). This model adaptively groups infants into like clusters (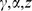) and implements a sparsity prior to prevent overfitting (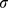).

Thus, we develop a graphical model (**Fig. 1**) for connecting hypothesized cognitive models to observed eye gaze data through formal linking hypotheses. On each trial of an experiment, an infant (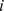) is exposed to some experimental stimuli (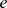) and produces observed eye movements (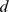). This observed gaze data is encoded as proportion of dwell time over a set of hypothesized areas of interest (AOIs). The inference framework discovers the set of underlying cognitive processes (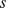) that operate on the stimuli to generate the observed data. This process is essentially a regression problem: Bayesian inference finds the relationship between predictor variables (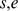) and observed outcomes (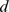). However, because gaze data is a distribution over AOIs rather than a single continuous variable, we connect these predictors and outcomes using a Dirichlet distribution (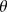) (see Graphical Model Details S1).

In the introduction, we identified three challenges to quantitative linking hypotheses: 1) multiple processes may drive eye-movements, 2) linking functions may be complex, and 3) a given sample of infants may be heterogeneous. This framework meets all three challenges. Because 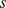 can encode any hypothetical cognitive model, the effect of multiple processes can be estimated simultaneously without forcing a dichotomy [16], [44]. However, if a process has little effect on observed eye movements, the prior on parameter values (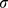) allows the model to discover this as well [45]. Second, the relationship between cognitive processes and observed eye movements need not be a simple linear one. As infants learn about novel objects, for instance, they may transition from no preference to a familiarity preference to a novelty preference [27], [29]. In this framework, any functional link can be encoded in the cognitive model (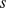). For simplicity, and to make minimal assumptions, we propose to do so through arbitrary degree polynomials [46], [47]. Here, again, the prior on model parameters (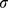) is used to discover the most parsimonious form of the linking function, penalizing complex polynomials.

Finally, infants in a sample may not come from a single homogenous group, but may actually represent two or more different groups, (e.g. slow and fast learners: [20], [48], [49]). This framework automatically and adaptively determines the number of groups of infants and the infants who belong to each group; each distinct group of infants may be best represented by a different cognitive model. The estimation of unique groups is performed using the Chinese restaurant process [50], [51], which has been used successfully to determine unique groups in adult experiments [52]. Clusters are discovered in this process by treating participants by analogy to customers in a Chinese restaurant. As each customer enters, he sits at each occupied table (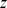) with probability proportional to the number of occupants, but also chooses a new table with some small probability (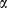). This implements a rich-get-richer scheme in which groups that account for the behavior of many infants become favored, and the most parsimonious number of groups is discovered. A hyper parameter (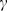) obviates a direct decision about the probability of choosing a new table.

In addition to tackling these difficult problems, this framework provides one more major advantage over traditional methods: all gaze data are treated as potentially relevant. Hypothesized cognitive processes should fit both training (or habituation) and test trials, off-screen looks should not be discarded, and side biases should not preclude infants from analysis [53], [54]. When all of these strengths are taken together, this framework can provide a much richer understanding of the processes that account for infant behavior (see e.g. [52], [55]). Using quantitative linking hypothesis in this framework, we can ask not only whether structure in the stimuli affected infant behavior, but also deeper questions about how and why this change took place. Credible intervals on the model parameters (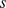) allow us to directly infer and describe the infant cognitive processes that we intend to study [56]. In the next section, we apply the model to data from a set of experiments investigating the role of attentional cues in infant learning [36].

### Case Study: Attentional Cues and Infant Learning

**Figure 2:**
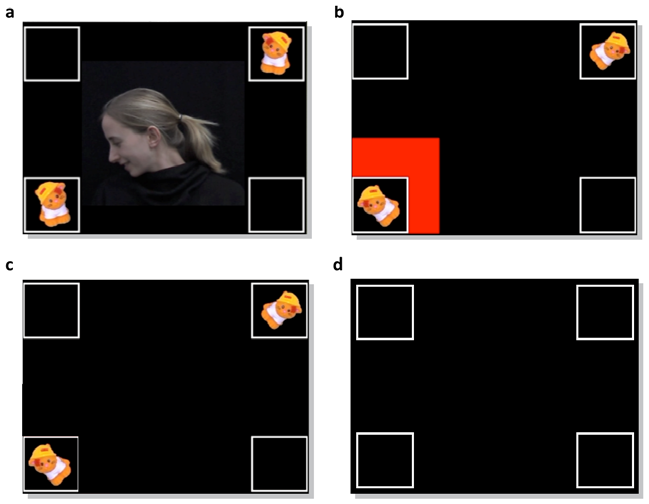
Training and Testing Trials. Training and testing trials from [36]. In the Face condition (a), a centrally-located face directed infants' attention to one of the boxes. In the Square condition (b), a red flashing square highlighted one of the boxes. In the No Cue condition (c) only the multi-modal regularity was present. On test trials (d), all boxes remained empty while infants heard one of the sounds from training. The actor in the photograph has given written informed consent, as outlined in the PLoS consent form, to publication of her photograph.

The previous section outlines a general framework for quantitative linking hypotheses that is applicable across a wide range of studies of infant cognition. In order to demonstrate its utility in a specific case, this section describes its application to a set of experiments investigating the role of attentional cues in infant multi-modal learning. In each of the experiments, 8-month-old infants were exposed to videos in which sounds and objects' on-screen locations were reliably related. When objects appeared in the top-left and bottom-right boxes, one sound was heard. When other objects appeared instead in the top-right and bottom-left boxes, a different sound was heard (**Fig. 2**). Subsequently, infants were exposed to test trials in which all four boxes were blank, but one of the sounds from training was played. If infants had learned the sound-location regularities, they were expected to preferentially attend to the locations that had co-occurred with each sound.

Wu and Kirkham [36] asked whether attentional cues might change the way that infants learn multi-modal regularities. In the Face condition (**Fig. 2a**), a female face appeared in the center of the screen, and turned to one of the lower boxes in which an object appeared. In the Square condition (**Fig. 2b**), a red flashing square instead highlighted the same box. Finally, in the No Cue condition (**Fig. 2c**), infant multi-modal learning was assessed in the absence of either attentional cue. Analyses of looking preferences on test trials showed that infants reliably learned the multi-modal regularity only in the presence of the Face cue. Thus, Wu and Kirkham [36] concluded that infants learn differently from social and non-social cues, and that the former can increase the likelihood of learning multi-modal regularities by 8 months of age.

These findings provide insight into the role of attentional cues in infant learning: different cues can have a very different effect. These findings also suggest a number of follow-up questions: is the difference between the two cues qualitative (e.g. one helps, the other does not), or is it a difference of degrees? Are infants homogenous in their response to the cues? If not, are infants who attend more strongly to the cues the same infants who show stronger multi-modal learning? Do infants orient attention to the Face in the same way that they orient attention to the Square? These questions might be addressed empirically in numerous follow-up experiments. However, it is possible that the answers reside in the current data but are opaque to common analytical tools (e.g. ANOVAs). In the following section, we formalize a set of quantitative linking hypotheses for these cued multi-modal learning experiments. With this richer analysis, we can leverage the existing data to answer questions about the mechanistic underpinnings of the observed differences in these experiments.

#### Quantitative Linking Hypotheses

To analyze the data from these experiments, we develop quantitative linking hypotheses for them in accord with the graphical model proposed above (**Fig. 1**). Thus, we specify formally the connection between the observed eye-movement data (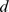), observable experimental conditions (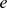), and the unobservable, hypothesized cognitive processes (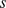). By analogy to regression, the data are the dependent variable, the experimental conditions are the independent variables, and the cognitive processes parameterize these independent variables. On each trial of the experiment – whether training or testing – infants saw a black screen containing four boxes, one in each corner of the screen (**Fig. 2**). Thus, we define five areas of interest (AOIs): one for each of the four boxes, and a fifth to capture all other looks (including off-screen looks). The total data (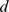) for an individual infant is thus the entire set of gaze proportions observed on each trial of the experiment. Formally, this is a matrix in which rows correspond to trials, columns correspond to AOIs, and each cell is the proportion of looking to a particular AOI on a particular trial. This whole matrix is the outcome to be predicted from the experimental conditions (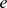) and the hypothesized cognitive processes (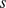).

Next we formally specify the experimental conditions to which infants were exposed on each trial. These are the observable variables through which the unobservable cognitive processes are hypothesized to lead to gaze patterns. While all four boxes were empty on test trials, on training trials two of the four boxes contained cartoon pictures of animals (**Fig. 2a–c**). These are coded with a binary indicator variable 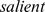 that specifies whether a box (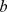) contains a picture. Further, in the Face and Square conditions (**Fig. 2a and 2b**), one of the boxes was highlighted by an attentional cue. We similarly define an indicator variable 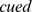 that specifies whether a particular box is cued.
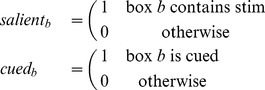
(2)


In addition to the visual stimuli, each trial also played a sound. We hypothesize that sounds do not directly affect looking preferences, but rather may alter looking patterns through the experience of learning sound-location contingencies (for evidence see [36] Experiment 6). In order to formalize this learning process (below), we encode each infant's experience with these contingencies in the experimental conditions (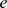). Thus, we also define the variable 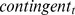 to encode an infant's cumulative looking proportion in a given box (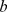) in the presence of a particular sound (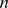) from trial 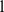 to trial 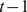. This looking history can then be used to predict looking on trial 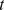. So, on trial 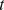 that plays sound 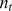 and on which the infant's proportion of looking in box 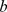 is 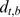, where 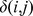 is Kroneckers delta function that returns 1 if its arguments are equal and zero otherwise:
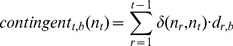
(3)


Last, we define the cognitive processes that act on these experimental condition variables to produce the observed gaze data. First, infants may have a baseline preference for some screen locations over others. For instance, significant proportions of infant participants are routinely excluded for exhibiting a bias for one side of the screen [53], [54]. Instead of excluding these infants, we include a preference constant 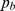 for each AOI in the model. This allows the contributions of the other variables to be considered once baseline preferences have been controlled.

Second, in accord with the experimental conditions described above, an infant's preference for a particular box may be altered by the presence of an object in that box (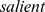), or the presence of a cue highlighting that box (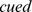). We let the strength of these factors be linearly scaled by parameters 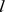 and 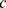 respectively. These function like slope terms in linear regression.

Finally, in these experiments, the question of interest is whether infants learn to associate sounds and objects/locations through co-occurrence. We thus define the effect of association between a sound and a location as a change in preference for that location over exposure to that contingency. More specifically, we let association between a sound and location be a function of time spent fixating that location in the presence of that sound (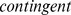). To avoid making apriori assumptions about the association function (e.g. that it is linear, or monotonic), we let 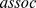 between box 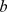 and sound 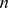 on trial 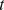 be an arbitrary degree polynomial function of cumulative looking time to 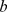 in the presence of sound 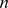. Since polynomials can approximate any functional form (e.g. splines [46], [47]), this is a general solution. As in testing for higher-order terms in standard regression, higher-order polynomial coefficients are pushed down to zero if they do not contribute to predictive power by the priors in the model (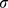). Equation 4 formalizes this definition, letting 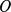 be the highest order non-zero term, and 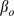 be the polynomial coefficient for each term 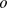.
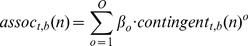
(4)


By formally specifying the observable experimental conditions and hypothesized cognitive process that act on these experimental conditions, we have specified quantitative linking hypotheses for the observed data. In summary, an infant's expected preference for each AOI 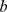 on trial 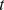 was modeled as an exponentiated linear combination of the above factors. The vector of preferences (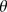) for all AOIs on trial 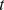 was passed through a Dirichlet distribution to predict the observed distribution of dwell time on that trial (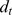). This is formalized in Equations 5.
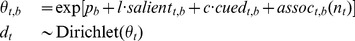
(5)


As in a regression analysis, we can now determine the quantitative effect that each of the hypothesized factors has on the pattern of eye-movements generated by each infant. We can use the differences in these parameters across conditions to understand whether and how different cues affect infant multi-modal learning. In order to determine the values of these parameters for each group of infants in each experimental condition, we perform Bayesian inference in the graphical model specified above using a Markov Chain Monte Carlo sampling algorithm. This sampling algorithm allows us to approximate the true distribution for each of these parameters, producing a set of credible intervals (similar to confidence intervals) that can be used to determine the likelihood that parameters are non-zero, as well as their likely range [57], [58]. Full technical details can be found in Inference Details S2.

Before we analyze the experimental data, however, we first present a set of simulation studies designed to demonstrate the robustness of the graphical model and the inference procedure. Because we propose a non-standard analytic framework, we must demonstrate that it behaves as expected. The simulations in the next section confirm that the inference procedure can recover correct parameter values when ground-truth is known.

## Methods

### Ethical Statement

All infant experimental procedures were approved by the School of Social Sciences, History and Philosophy Ethics Committee at Birkbeck, University of London (protocol 2324). Informed consent was acquired in writing from the parents of all infants.

### Simulations

While this framework is built on well-established theoretical principles, it is still critical to certify empirically that it behaves as expected [59]. Thus, we first validate the analysis empirically in a set of simulation studies. Recall that this analysis works by specifying a model that generates the observed data and then inferring its parameters. We can test this inference process by generating data from a model using known parameters. If the inference process works properly, we should be able to recover these same parameters.

We considered three impediments to applying quantitative linking hypotheses to infant looking data: 1) the possibility of multiple groups of infants, 2) the contribution of multiple factors, and 3) the potential for non-monotonic linking functions. The following simulations show the framework's capacity to solve all three of these problems. In each analysis, we expose simulated infants to a series of trials comparable to those seen in the Face and Square conditions.

#### Simulation 1

Developmental researchers typically use differences in eye-gaze behavior at different ages to understand how cognitive processes develop [60], [61], but stable group differences can be found even at a single age [48], [49]. In Simulation 1, we tested the analysis on data generated from a mixture of a known number of groups. In all cases, the analysis robustly determined the correct number of groups and clustered infants correctly.

Infants in Wu & Kirkham's [36] study were simulated by constructing training and testing trials identical to those in the original experiments. Simulated infants were exposed to four consecutive blocks each consisting of six training trials and a test trial. On each training trial, objects appeared in two of the boxes (top-left and bottom-right, or top-right and bottom-left), and the lower box was cued. The appearance of objects in each configuration also co-occurred on each trial with a sound unique to that configuration. Each of the two configurations was seen three times in each block of training trials, and order was pseudo-randomized within a block. After all six training trials, infants saw one test trial on which the screen was empty, but one of the two sounds was heard. These seven trials together comprised one block, and simulated infants were exposed to four blocks total. Each sound was tested twice across the four test trials.

Thirty simulated infants were generated for each of four group numbers (1, 2, 3, and 4) 30 times. On each run for a particular group size, the number of infants in each group was determined by a draw from a Multinomial distribution with an equal probability for each group. For instance, for group size 3, the number of infants in each group was drawn from 
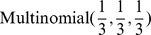
. Parameters for each group were assigned by drawing values without replacement from 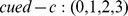, 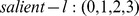, and 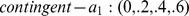. Thus, all true association functions were linear. Baseline preferences for each AOI were assigned by drawing values uniformly from 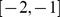 for each on-screen location and from 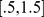 for the off-screen location. Values were chosen to be comparable to those found in analyses of the real experimental data (below), and to ensure that groups within a run were sufficiently different. Inference on each run was performed by sampling 1,000 times for each individual infant and then 5,000 times for all infants together. The first 2,500 samples of the group chain were discarded to ensure sufficient burnin (See Inference Details S2 for details of the MCMC sampling algorithm).

#### Simulation 2

Developing quantitative linking hypotheses for eye movement data is difficult partly because multiple cognitive processes are likely to contribute to the observed data [1], [21], [22]. In the previous section, we considered three potential contributors: 1) preference for boxes containing objects (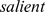), 2) preference for cued boxes (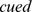), and 3) learning sound-location co-occurrence regularities (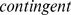). In Simulation 2, we parametrically manipulated the contribution of each factor to simulated infant gaze data, and showed that correct values could be recovered through inference.

Individual infants were exposed to training and testing trials identical to those described in Simulation 1. What varied was simulated infants' sensitivity to cues, salience, and rates of associative learning. Six unique values were chosen for each parameter in half-steps compared to the steps in Simulation 1: 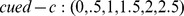, 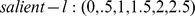, and 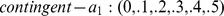. As in Simulation 1, all association functions were linear. Baseline preferences for each AOI were again assigned by drawing values uniformly from 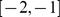 for each on-screen location and from 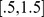 for the off-screen location. Each possible combination of 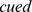, 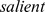, and 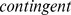 parameters was tested once, resulting in 216 total simulations. Each simulation was run with 10 simulated infants in one group. Inference was performed by sampling 2,000 times for each individual infant and then 50,000 times for all infants together. The first 5,000 samples of the group chain were discarded to ensure sufficient burnin.

#### Simulation 3

The previous simulations show that our framework can successfully recover correct association functions when the true functions are linear and have a positive slope, i.e. when increased learning leads to increased looking [49]. This linking hypothesis is implicit in many studies of infant learning, but it is far from the only one employed. Often, increased learning is hypothesized to lead to decreased learning, as in habituation [2], [5], [62]. But sometimes the function linking looking and learning is proposed to be more complex. For instance, Hunter and Ames [27] argued that the function may be non-monotonic, with learning leading first to increased looking and subsequently to decreased looking (see also [29], [30]). In Simulation 3, we generate data from true models with four kinds of learning functions: 1) linear increasing, 2) linear decreasing, 3) u-shaped up, and 4) u-shaped down. We show that inference can recover all four kinds successfully.

Individual simulated infants were exposed to training and testing trials identical to those described in Simulations 1 and 2 above. For each simulation, parameters for 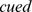 and 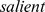, as well as baseline preferences were chosen randomly with replacement from the same set of values as in Simulation 2. In this simulation, we manipulated the *associative learning* functions used to generate the data. Two functions encode simple linear linking hypotheses: 1) learning increases looking (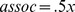), and 3) learning decreases looking (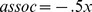). Two additional functions encoded non-monotonic linking functions: 1) learning leads first to increased and then decreased looking (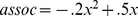), and 2) learning leads first to decreased and then increased looking (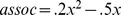). Thirty simulations were run for each of these possible learning functions with 10 infants in each run. Inference was performed by sampling 2,000 times for each individual infant and then 50,000 times for all infants together. The first 5,000 samples of each group chain were discarded to ensure sufficient burnin.

### Experiment

Having validated the proposed framework on simulation data, we apply the quantitative linking hypotheses proposed above to data from three experimental conditions in [36]. Instead of comparing the effects of different attentional cues using raw test preferences, as in standard analyses (e.g. ANOVAs), inferring cognitive model parameters for each condition lets us analyze the effects of different cues directly on attention and learning. Infants were exposed to two different kinds of cues as well as a no cue baseline condition. However, because the cues can be encoded in the same linking hypotheses, their effects can be compared directly as quantitative changes in attention and learning parameters (see also, [63]).

In each condition, infants were exposed to a series of training trials in which two objects appeared in opposite diagonal boxes on the screen (**Fig. 2**). When objects appeared in the top-right and bottom-left boxes, one sound was heard. When objects appeared instead in the top-left and bottom right boxes, a different sound was heard. Each condition consisted of four blocks of six such training trials. Within a block, each of the two location-sound regularities occurred an equal number of times in pseudo-random order. After six training trials, infants saw one test trial on which they heard one of the sounds from training, but all of the on-screen boxes were empty (**Fig. 2d**). In addition to this common design and procedure, infants in each condition were exposed to a different attentional cue during training trials. In the Face condition, an on-screen face appeared and turned to look at the lower on-screen object(**Fig. 2a**). In the Square condition, a flashing red square surrounded the lower on-screen object (**Fig. 2b**). Finally, the No Cue condition, in which no attentional cue was present, served as a baseline for comparison (**Fig. 2c**). Inference for parameters was performed for 26 8-month-olds in the No Cue condition, 29 8-month-olds in the Face condition, and 30 8-month-olds in the Square condition (see [36] for full participant details).

Linking hypotheses were defined for each condition as described above. In the No Cue condition, the value of the 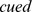 indicator function was set to 0 for each AOI on each trial. Instead of excluding it apriori, this acts as a further test of the model priors in regularizing non-contributing parameters. Inference for model parameters was performed separately for each experimental condition.

## Results and Discussion

### Simulations

#### Simulation 1

**Figure 3:**
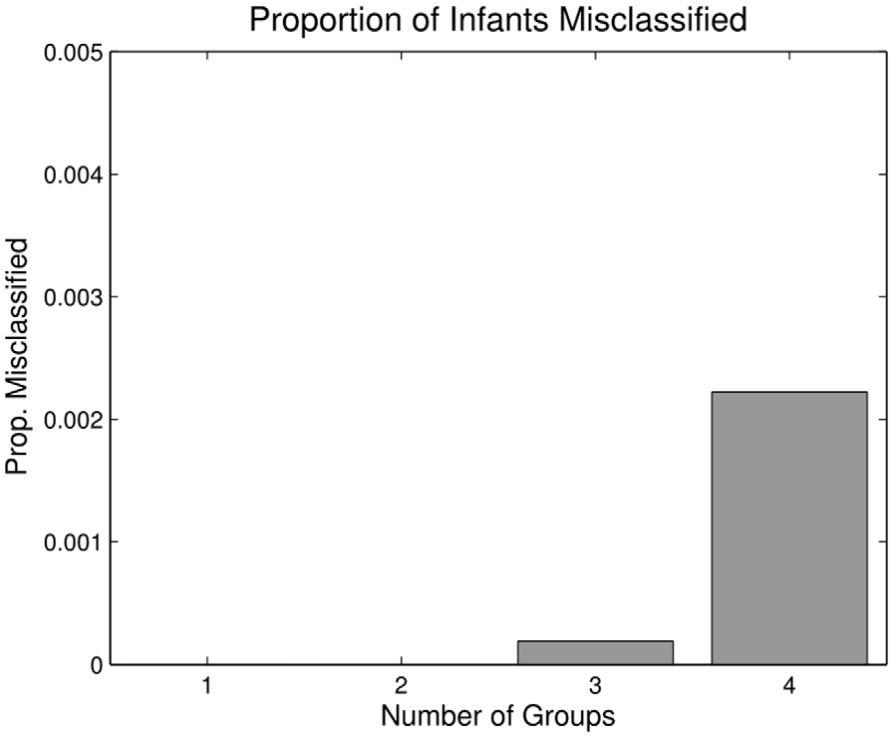
Infants Misclassified in Simulation 1. Proportion of infants misclassified in Simulation 1. As less than a quarter of one percent of infants were assigned to the wrong group in the worst case, we can be reasonably sure that the inference process is robust.

Across all 120 simulations (30 runs at each of the four group sizes), the correct number of clusters was identified in all but one. On one run at group size 4, the analysis identified only three clusters. Nonetheless, it is possible that even if the correct number of clusters was identified, the proportion of infants in each cluster was incorrect. To determine the proportion of infants misclassified, we computed the number of infants assigned to an incorrect group on each sample from the posterior distribution (see [64] for a derivation of this distance metric). The number misclassified was averaged across all 1,000 posterior samples for each run, and the 30 runs for each group size were averaged together. **Fig. 3** shows the average proportion of infants assigned to the wrong group at each group size. Group assignment was perfect when the number of true groups was 1 or 2, and less than a quarter of one percent (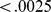) of infants were misclassified at the higher group numbers. These results clearly show that the analysis is capable of dealing with heterogeneous groups of infants.

#### Simluation 2

**Figure 4:**
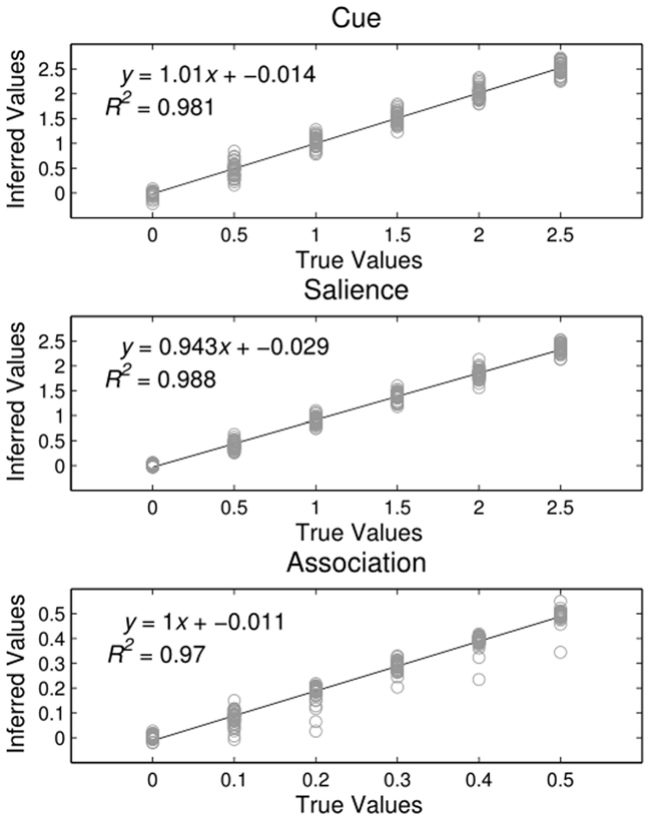
Parameter Values for Simulation 2. Best fit lines for true and inferred parameter values for each of the three factors hypothesized to affect infant gaze patterns in the experimental data. Inference for parameter values proved to be highly reliable.

Across all factors, correct parameter values were recovered well at each ground-truth level (**Fig. 4**). In general, when parameters were estimated incorrectly, this was due to underestimation, as evidenced by the negative constant in each graph of **Fig. 4**. This suggests that the Type I error rate should be low. The poorest estimation occurred in the case of inferring values for the association parameter 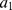. True positive values were particularly likely to be underestimated when the values of other parameters were zero. That is, when infants' initial preferences were unaffected by experimental conditions, and were thus more uniform, changes in preferences due to learning were more difficult to pick up. Nonetheless, the high 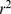 for best-fit lines for each factor (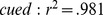, 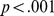; 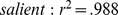, 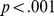; 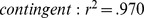, 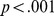) indicate that inference was successful in recovering true parameter values.

#### Simulation 3

**Figure 5:**
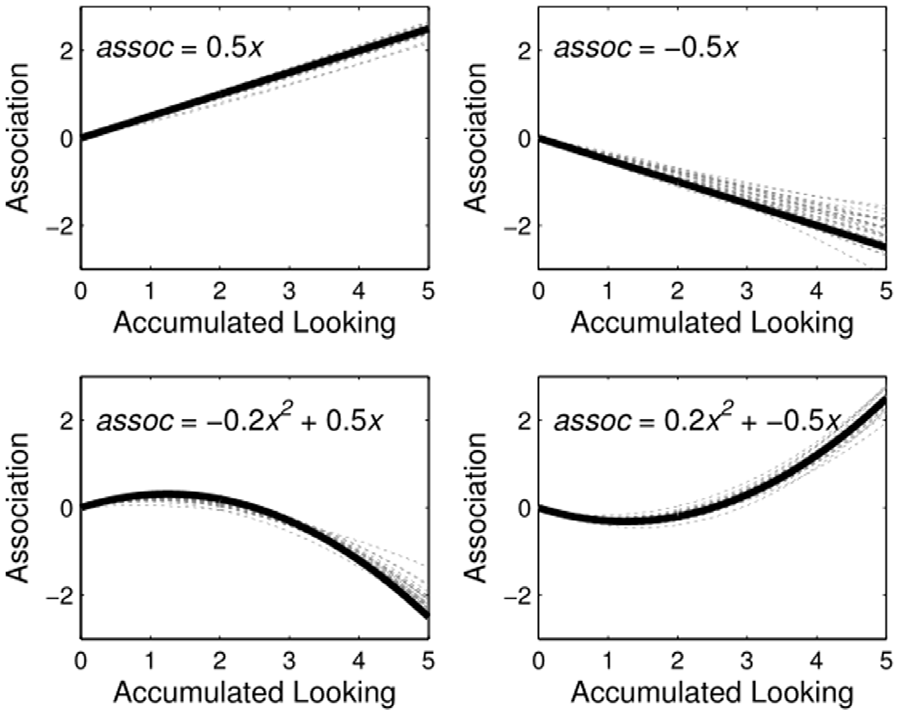
Learning Functions in Simulation 3. True functions (solid black) and 30 inferred functions (dashed gray) for each tested kind of learning function. The inference process was quite successful in recovering the properties of the true generating functions.

**Fig. 5** shows the true learning function and 30 inferred functions for each condition. To determine how well the inference process found non-linear functions when they were true, and rejected nonlinear functions when they were not true, we examined the 95% credible intervals for parameters generated for each function type. When the true parameter for a function was non-zero, the 95% credible interval should correspondingly not cross zero. If the interval did cross zero, this would be a Type II error. In contrast, when the true parameter value for a function was 0, the 95% credible interval should cross zero. If this was not the case, we would have made a Type I error. Because extensive sampling is computationally expensive, we added a .001 buffer around zero. Table 1 shows the proportion of simulations run for each learning function for which each of the two association parameters (linear – 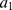, quadratic – 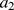) were found to be nonzero. Discrimination was perfect for the quadratic term, indicating that the inference process can find u-shaped learning functions when they are the true generating functions. Further, the Type II error rate was also within acceptable margins. Only on 2.5% of all simulations did the 95% credible interval for the linear parameter overlap zero.

**Table 1:** Non-Zero Learning Parameters in Simulation 3.

Learning Function	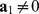	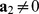
Linear Positive: ( 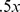 )	1.0	0
Linear Negative: ( 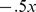 )	.933	0
U-shaped up: ( 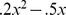 )	.967	1.0
U-shaped down: ( 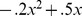 )	1.0	1.0

Proportion of association parameters estimated to be nonzero for each learning function in Simulation 3.

#### Simulation Discussion

Thus, in three simulations, we validated the model and inference process in experimental settings like those in the empirical data of interest. In Simulation 1, we showed that this analysis finds the correct number of clusters when infant participants are heterogeneous. In Simulation 2, we showed that correct quantitative values can be recovered for the hypothesized cognitive processes, even when multiple processes interact to produce the observed eye movements. Finally, in Simulation 3, we showed that this analysis can recover non-monotonic learning functions when they are correct, and can avoid positing complex learning functions when they are incorrect. These simulations license the application of the proposed framework on experimental data.

### Experiment

**Figure 6:**
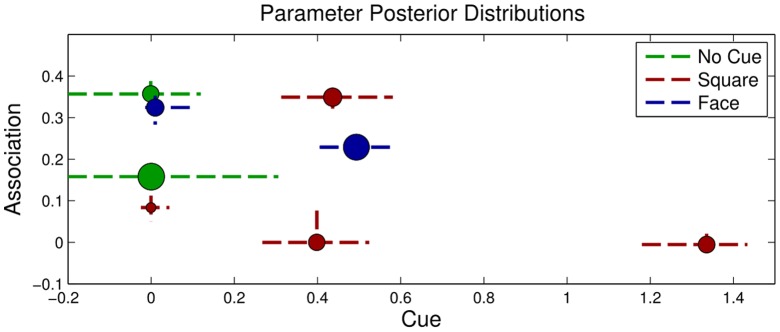
Parameters Inferred for Empirical Data. Posterior distributions for cue (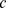) and association (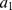) parameters for infants from [36]. Each circle indicates a cluster, and its size indicates the proportion of infants in that condition in that cluster. Circles are centered at median parameter values, and dashed lines indicate 68% credible intervals, akin to 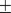
1 SE.

Having validated the graphical model framework in three simulations, we apply it to empirical data from Wu & Kirkham [36]. Inference yields full posterior distributions for all cognitive model parameters, estimating the contribution of each factor in the context of all other factors. However, because the questions of interest relate specifically to attention and learning, we focus on two key factors: attention to the cue (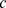) and the association function (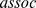). The other factors – preference for particular screen locations (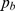) and preference for boxes with stimuli (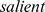)– work to reduce noise in analyzing these key factors. **Fig. 6** shows estimated parameter values for both factors for infants in each experimental condition.

We first note that in no condition was the sample of infants best described coming from a single homogeneous group. Two distinct groups were identified in the Face and No Cue conditions, and four groups were found in the Square condition. Thus, even within one condition, infants learned and used cues differently. Second, all learning functions were linear; credible intervals for all association coefficients 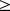
2 overlapped 0 in all conditions. Thus, **Fig. 6** shows the first-order association coefficient (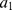) for each group.

Next we consider each condition in turn, letting the No Cue condition be our baseline for comparison. The sample of infants in the No Cue condition was best described as coming from two clusters: The first cluster, accounting for 72.3% of infants across MCMC samples, was characterized by slow learners, having a median association coefficient (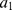) of .158. The smaller cluster, accounting for the remaining 27.7% of the infants across MCMC samples, described faster learners, having a median association coefficient of .357. In their original analysis, Wu and Kirkham [36] did not find reliable evidence of learning on average for infants in this condition. Our new analysis likely found evidence of learning for two reasons. First, the analysis in [36] considers infants' looking preferences on only the four test test trials, but the model-based analysis is informed by looking behavior on all 28 experimental trials for each infant. Further, more than 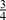 of the infants in this sample were found to be relatively slow learners, and thus a less sensitive analysis showing learning would have to be carried by a small proportion of the infants. Finally, we note that the median cue parameter (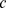) values for both clusters were 0. This result shows that in real empirical data, this modeling framework can correctly discover parameters that do not contribute to prediction of gaze patterns, avoiding Type I errors.

When 8-month-old infants encountered the same multi-modal regularities in the presence of a social cue, their learning behavior was reliably different. As in the No Cue condition, infants in the Face condition were best described by two clusters. The first, accounting for 69.4% of the sample, contained infants who learned more slowly (median 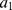 = .229) and whose attention was directed by the cue (median 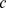 = .493). The second cluster, accounting for 30.6% of the sample, contained infants who learned more quickly (median 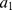 = .324) and whose attention was not directed by the cue (median 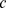 = .009). Thus, counter-intuitively, those infants who responded most to the cue did not learn as quickly; the fast learners in the Face condition learned at the same rate as the fast learners in the No Cue condition (as seen in their overlapping 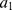 credible intervals). However, the slower learners in the Face condition did learn more quickly than the slow learners in the No Cue condition. Thus, the Face cue accelerated learning for the large group of slower-learning infants.

Infants cued to these same multimodal regularities by a red flashing square fell into four distinct clusters. The first cluster, accounting for 33.9% of the sample, contained infants who learned quickly (median 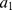 = .349), and whose attention was directed by the red square (median 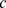 = .436). A second, small cluster accounting for 10% of the sample consisted of slow learners (median 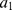 = .084) whose attention was not directed by the cue (median 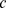 = 0). Finally, the sample also contained two clusters of non-learners (median 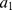 = 0 and −.005), accounting for 27.8% and 28.2% of the sample respectively. The attention of infants in both groups was directed by the cue, the second more strongly than the first (median 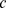 = .398 and 1.33). Thus, the Square condition contained a small cluster of infants who learned just as quickly as in previous conditions, but the remaining 66.1% of the infants learned more slowly than any of the infants in the previous conditions, and over half of the infants showed no learning at all. Thus it appears, as Wu and Kirkham [36] suggested, that the cue competed with the regularity for attention, and even those infants who resisted the draw of the square learned more slowly. Even the gaze of the fastest learners, in contrast to those in the Face condition, was drawn by the cue. Perhaps these fast learners were able to learn in spite of the cue rather than because of it, as seen in the Face condition?

#### Experiment Discussion

Not only do these results confirm the main findings from Wu and Kirkham's [36] coarser analysis, they also provide deeper insight into how attentional cues guide (or interfere with) infant multi-modal learning. First, they provide clear evidence that not all infants respond to attentional cues in the same way. Within each cue condition, infants were best described by multiple clusters, some driven more by attentional cues than others. Second, they show that individual infants learn at different rates, and that infants who use attentional cues are not always those who learn fastest. For instance, the addition of the Face cue did lead to improved learning in general relative to the No Cue condition, but it did so exclusively for slower learners. Also, infants who attended most strongly to the Square cue showed no evidence of learning at all. Thus, even when cues are reliable, they may not accelerate infant learning; in some cases they may even inhibit it.

Finally, we note that these results show evidence of learning in conditions in which it was not found in the analysis reported in [36]. This greater sensitivity is likely due to three contributing factors. First, the analysis in [36] considers infants' looking preferences only on test trials – a small fraction of the data. In contrast, this model-based analysis infers underlying cognitive processes that account for all of the available looking data. Second, the analysis in [36] assumes that infants in each sample come from one homogenous group. However, the analyses here show that this may be incorrect, and that better conclusions can be drawn by separating infants into distinct clusters [31]–[33]. Finally, the analysis in [36], and in the majority of other infant experiments, is performed at the level of raw looking preferences. Thus, underlying learning processes may be hidden by other processes that also control eye movements. This model-based analysis isolates the contribution of the variables of theoretical interest, yielding greater power to detect their effects.

## Conclusion

Infancy researchers have made tremendous progress by using eye gaze data to ask questions about early cognition and development. The majority of this work has used qualitative linking hypotheses, but we propose that even faster and more rigorous progress can be made through model-based analyses using quantitative linking hypotheses [1], [4]. In addition to providing insight into cued attention and learning, the present analyses also have potential implications for two more general issues raised in the introduction. We follow the discussion of these issues with a conclusion about possible extensions of this framework.

### Competing Hypotheses

One strength of quantitative linking hypotheses is that they facilitate direct comparison of competing theories for the same data. In the previous sections, we argued that changes in looking preferences over the course of these experiments arise from associations between heard sounds and fixated locations, and modeled this learning with the 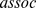 function. Alternatively, preferences could change over time through habituation; infants' preferences could change as a function of looking to a location independent of the concurrent sound. For instance, Wu and Kirkham [36] speculated that infants in the Square condition may have learned a general preference for the cued locations even though they did not learn specific sound-location relations. This hypothesis can be tested directly against the association hypothesis by encoding both in 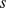 and examining the posterior parameters.

Thus, we introduce a habituation function to encode learning a preference for fixated locations independent of the sounds being heard. This 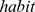 function operates like the 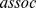 function, being an arbitrary degree polynomial function of cumulative looking time to a particular location (Equation 6). However, when this function was included in the cognitive model for each condition, 95% credible intervals for 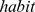 coefficients overlapped 0 in all cases. Thus, quantitative linking hypotheses can be used to test competing accounts for the same data. This type of analysis could have the potential to resolve some of the “rich” vs. “lean” arguments in the infant literature [12]–[14].
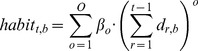
(6)


### Non-monotonic Learning Functions

Several theoretical accounts of infant learning posit that infants' preferences for a stimulus may change non-monotonically over the course of exposure; infants may show first a familiarity preference and then a novelty preference [27]–[29]. Thus, the framework presented in this paper encodes learning functions as arbitrary-degree polynomials, allowing them to approximate any functional form. Further, Simulation 3 showed that the inference procedure can correctly recover non-monotonic learning functions when they are appropriate for the data. However, no such functions were found in the analysis of the empirical data from [36]. Why?

One possibility is that non-monotonic linking functions arise in a different kind of experiment or at a different age. For instance, the infants analyzed may simply have not had enough time to pass through the familiarity-preference portion of the learning function into the novelty-preference portion [27]. This hypothesis cannot be ruled out conclusively by the present data. We propose, however, an alternative possibility. It may be that in some cases, apparent non-monotonic linking functions may arise from differences in baseline preferences for different stimuli.

The analyses above include a set of baseline preference parameters (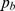) to control for infants' apriori preferences for different locations on the screen. When these parameters were included, none of the higher-order coefficients for the 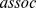 functions were found to be nonzero. However, when baseline preference parameters were not included, non-monotonic learning functions were found in both the Face and No Cue conditions. Consequently, we propose that, at least in some cases, observation of non-monotonic linking functions could be an artifact of different baseline preferences.There could, of course, be cases in which true non-monotonic learning functions arise. This framework provides one approach for documenting them.

### Extending the Framework

The framework presented in this paper was designed to infer cognitive processes from eye gaze data in which the data of interest are a pattern of dwell times over a set of areas of interest (AOIs). Consequently, the cognitive model (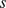) and experimental settings (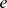) are connected to the observed gaze data (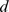) by means of the Dirichlet distribution (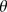). However, if the data of interest were in a different form – for instance if the critical question was about latencies rather than dwell times – a different linking function could be used. For such data, a Normal or Exponential distribution may be more appropriate. Such a model would still benefit from the adaptive clustering and parameter regularization offered by this graphical model framework.

We note also that recent years have seen fervent arguments about the relative merits of Bayesian approaches to cognition [65]–[67]. The analysis presented here is agnostic about these issues; Bayesian data analysis is a statistical technique requiring no commitment to any particular framework for modeling cognition [56], [58]. In fact this paper describes a simple associative model. This is an explicit strength of the framework advocated here: any cognitive model that can be characterized formally can be encoded in the hidden variable vector 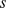, allowing competing models to be compared directly. While quantitative linking hypotheses have been proposed for specific experiments (e.g. [49], [62]), this paper presents a general framework applicable to many eye movement experiments, as well as for other indirect behavioral measures. Thus, we hope this framework will facilitate asking and answering future questions about early cognitive processes and their development.

### Software Package

Software for all simulations reported in this paper is available on the first author's website. This software is free and open source, but was written in MATLAB R2009b, and thus relies on this proprietary software.

## Supporting Information

Graphical Model Details S1 (PDF)

Inference Details S2 (PDF)
