# Supplementary material for: Quantitative Linking Hypotheses for Infant Eye Movements
Source: PLoS One. 2012 Oct 26;7(10):e47419. doi: 10.1371/journal.pone.0047419 (PMC3482231; doi:10.1371/journal.pone.0047419)
Supplement: Graphical Model Details S1 — (PDF) [file pone.0047419.s001.pdf]

Graphical Model Details S1

The model framework presented in **Fig. 1** is known in statistics as a Dirichlet process mixture model [1, 2]. In this model, the data observed on each experimental trial are generated by a Dirichlet distribution, but the Dirichlet's parameters can vary across infants. The hypothetical population of infants is modeled as a mixture of an unknown number of discrete groups of unknown size, and each group has different parameters. Each sample of infants, as observed in an experiment, is modeled as draw from this mixture. Thus, the infants in each experiment represent a mixture of different groups, with a prior preference for fewer, larger groups. Bayes rule (Equations 1) is used to infer, from the data and from prior constraints, a distribution of beliefs for these model parameters.

This model has several equivalent formulations, each suited for different inference algorithms (see S2). In this paper, we use the Chinese restaurant process (CRP) formulation, which allows group identity and group parameters to be inferred separately [2, 3]. In the Chinese Restaurant Process, groups are conceptualized as tables in a Chinese restaurant, and infant participants as customers. When customers enters the restaurant, they choose a table (z) in proportion to the number of customers already at that table. This yields a rich-get-richer clustering scheme. However, with some small probability (α), a customer chooses a new table, starting a new group. A property of this process called *exchangeability* allows each customer to be treated as the last customer, producing a proper probability distribution. For a more detailed tutorial, see [4]. In order to avoid specifying a particular concentration parameter (α), we let this parameter be drawn from an Exponential distribution. This formally encodes a prior preference for fewer clusters, but lets the data decide the strength of this prior. This Exponential distribution also has a parameter (γ) that we set to 1 in the simulations in this paper. In hierarchical models, the higher level at which a parameter is fixed, the more insensitive the posterior distribution is to the specific value of that parameter. Equations S1 formalize this portion of the model.

$$\begin{aligned} \alpha &\sim \text{Exponential}(\gamma) \\ z &\sim \text{CRP}(\alpha) \end{aligned} \tag{S1}$$

In order to infer a cognitive model for each infant's gaze behavior, we formalize the data observed on each trial as a distribution of dwell times over a set of areas of interest (AOIs). Formally, let the AOIs in an experiment be defined as the vector A, and suppose that the infant is exposed to t experimental trials. Then, $d_{i,t'}$, the data for infant i on trial t' is a length $|A|$ vector of proportions that sums to 1. Consequently, d_i, all of the data for infant i, is a $t \times |A|$ matrix in which all of the trials are concatenated vertically. This data is modeled as being generated by draws from t Dirichlet distributions with parameters $\theta_{i,t}$, a matrix of size $t \times |A|$ that encodes our prior belief for the likely dwell time distribution over AOIs on each trial t. This matrix θ is a product of two separate components: e_i –

the experimental settings that infant i sees on each trial, and s_z – the cognitive model parameters for group z of which infant i is a member. These two components function like the predictors and weights in a regression model respectively.

In any experiment, we can imagine an arbitrary number of factors that may contribute to the observed distribution of gazes. These might include infants' familiarity with the objects in each AOI, the visual properties objects, relationships of these objects to co-occurring audio stimuli, how long infants have been in the experiment, etc. Let r be a vector of such factors, the elements of which work like predictor variables in standard linear regression. Any experimental trial can then be described as having some value for each of these factors for each area of interest. We encode this information as $e_{i,t'}$, the $|A| \times |r|$ matrix containing the value for each of these predictive values for each AOI that infant i sees on trial t'. The matrices for all trials can then be concatenated to produce a $t \times |A| \times |r|$ matrix. This matrix (e_i) is then used to predict the gaze patterns seen on each trial (d_i).

Thus, as in regression, the preference for each AOI is produced through a weighted linear combination of predictors r. Bayesian inference in this model discovers the weight for each of these factors for each group of infants (s_z). In order to make the model as general as possible, we let each of these weights be any continuous value in the range $(-\infty, \infty)$. Thus, some factors could contribute positively to looking, others could contribute negatively, and some could not contribute at all. In accord with Ockhams razor, we would prefer not to include predictors in the model if they do not contribute significantly to the prediction of gaze data. To do this, we put priors on the parameters in s_z, letting them be drawn from a normal distribution with mean 0 and variance σ^2. This encodes a prior preference for 0-valued parameters, but does not yet specify the strength of this preference. As we did in determining the number of groups, we put a hierarchical prior on σ to let the data decide the strength of our preference for sparsity. We use a Jeffreys prior [5], shown in previous work to work well in regularizing regression coefficients [6]. We approximate the Jeffreys prior by drawing σ from a Gamma distribution with very small shape and rate parameters. Finally, because the weights in s_z can take any continuous value, but the Dirichlet distribution connecting predictors to outcomes must have non-negative parameters, we exponeniate the products of weights and predictors. This portion of the model is formalized in Equations S2.

$$\begin{aligned} \sigma &\sim \text{Gamma}(\epsilon, \epsilon) \\ s &\sim \text{Normal}(0, \sigma^2) \\ \theta &= \exp[s_z \times e] \\ d &\sim \text{Dirichlet}(\theta) \end{aligned} \tag{S2}$$

References

1. Antoniak CE (1974) Mixtures of Dirichlet processes with applications to Bayesian nonparametric problems. Annals of Statistics 2: 1152–1174.

2. Rasmussen CE (2000) The infinite Gaussian mixture model. Advances in Neural Information Processing Systems 12: 554–560.

3. Aldous D (1985) Exchangeability and related topics. In: École d'été de probabilités de Saint-Flour, XIII–1983, Berlin: Springer. pp. 1–198.

4. Goldwater S, Griffiths TL, Johnson M (2009) A Bayesian framework for word segmentation: Exploring the effects of context. Cognition 112: 21–54.

5. Jeffreys H (1961) Theory of probability. Oxford, England: Oxford University Press, 470 pp.

6. Figuerido MAT (2002) Adaptive sparseness using Jeffreys prior. Advances in Neural Information Processing Systems 14: 722–729.
