# Supplementary material for: Quantitative Linking Hypotheses for Infant Eye Movements
Source: PLoS One. 2012 Oct 26;7(10):e47419. doi: 10.1371/journal.pone.0047419 (PMC3482231; doi:10.1371/journal.pone.0047419)
Supplement: Inference Details S2 — (PDF) [file pone.0047419.s002.pdf]

Inference Details S2

This graphical model described in Graphical Model Details S1 and **Fig. 1** defines a distribution that assigns a likelihood to every possible set of observed data given a particular set of parameters s. Using Bayes rule (Equations 1), we can invert this likelihood in order to determine the probability of different model parameters given the observed data. However, because we cannot derive this distribution analytically, we must approximate it with a Markov-Chain Monte Carlo sampling algorithm. Because the data are distributed according to a Dirichlet distribution, but model parameters are distributed according to the non-conjugate Normal distribution, we use a combination of Metropolis-Hastings (MH) steps for sampling cluster parameters and Split/Merge steps for sampling cluster assignments [1, 2]. In all of the analyses in this paper, we alternated 5 MH steps with 1 Split/Merge step. In Metropolis-Hastings steps, each proposal involved changing one model parameter in each cluster by a value drawn from a Normal distribution with mean zero and fixed variance. This variance was tuned for each simulation so that the sampling acceptance rate would be approximately 23% [3]. In addition to proposing new cluster parameters, each Metropolis-Hastings step also sampled a new cluster identity for each participant. Each participant was in turn removed from his/her current cluster, and probabilistically assigned to one of the current clusters or to one of two temporary auxiliary clusters according to the Chinese restaurant process distribution [1]. Auxiliary clusters, whose parameters are sampled from the prior distribution, help the sampler to explore larger areas of parameter space.

However, because Metropolis-Hastings steps alone are slow to create new clusters or to merge existing clusters, one Split/Merge step was attempted between every 5 MH steps. Each such step was randomly, uniformly chosen to be either a Split or a Merge step. In Split steps, the sampler considered a random partition of an existing cluster into two new clusters, one with the old parameters and one with all parameters adjusted as in the Metropolis-Hastings steps. The Merge steps considered collapsing two existing clusters into a single cluster. Proposal distributions are described in detail in [2].

Each simulation and analysis began by initializing a single cluster for each participant with parameters drawn from the prior distribution. After initialization, 2000 Metropolis-Hastings steps were performed for each individual participant, and the maximum-likelihood parameterization was used as the start for the group sampler. This helped to start the sampler in a high-probability area of parameter space and to reduce time to burn-in. Because learning was modeled with arbitrary degree polynomials, but denser sampling is possible when the model has fewer parameters, we started each learning function as a 4th degree polynomial and progressively reduced the order if the credible interval for coefficients overlapped zero in all cases. Finally, we used thinning to reduce correlation among samples, keeping only every 5th sample [4].

References

1. Neal RM (2000) Markov chain sampling methods for Dirichlet process mixture models. Journal of Computational and Graphical Statistics 9: 249–265.

2. Jain S, Neal RM (2007) Splitting and merging components of a nonconjugate Dirichlet process mixture model. Bayesian Analysis 2: 445–472.

3. Roberts GO, Gelman A, Gilks WR (1997) Weak convergence and optimal scaling of random walk metropolis algorithms. The Annals of Applied Probability 7: 110–120.

4. Raftery AE, Lewis SM (1996) Implementing MCMC. In: Gilks WR, Spiegelhalter DJ, Richardson S, editors, Markov Chain Monte Carlo in Practice, London: Chapman and Hall. pp. 115–130.
